# Structures of replication initiation proteins from staphylococcal antibiotic resistance plasmids reveal protein asymmetry and flexibility are necessary for replication

**DOI:** 10.1093/nar/gkv1539

**Published:** 2016-01-20

**Authors:** Stephen B. Carr, Simon E.V. Phillips, Christopher D. Thomas

**Affiliations:** 1 Research Complex at Harwell, Rutherford Appleton Laboratory, Harwell Oxford, Didcot, Oxfordshire OX11 0FA, UK; 2 Astbury Centre for Structural Molecular Biology, University of Leeds, Leeds LS2 9JT, UK

## Abstract

Antibiotic resistance in pathogenic bacteria is a continual threat to human health, often residing in extrachromosomal plasmid DNA. Plasmids of the pT181 family are widespread and confer various antibiotic resistances to *Staphylococcus aureus*. They replicate via a rolling circle mechanism that requires a multi-functional, plasmid-encoded replication protein to initiate replication, recruit a helicase to the site of initiation and terminate replication after DNA synthesis is complete. We present the first atomic resolution structures of three such replication proteins that reveal distinct, functionally relevant conformations. The proteins possess a unique active site and have been shown to contain a catalytically essential metal ion that is bound in a manner distinct from that of any other rolling circle replication proteins. These structures are the first examples of the *Rep_trans* Pfam family providing insights into the replication of numerous antibiotic resistance plasmids from Gram-positive bacteria, Gram-negative phage and the mobilisation of DNA by conjugative transposons.

## INTRODUCTION

**Figure 1.**
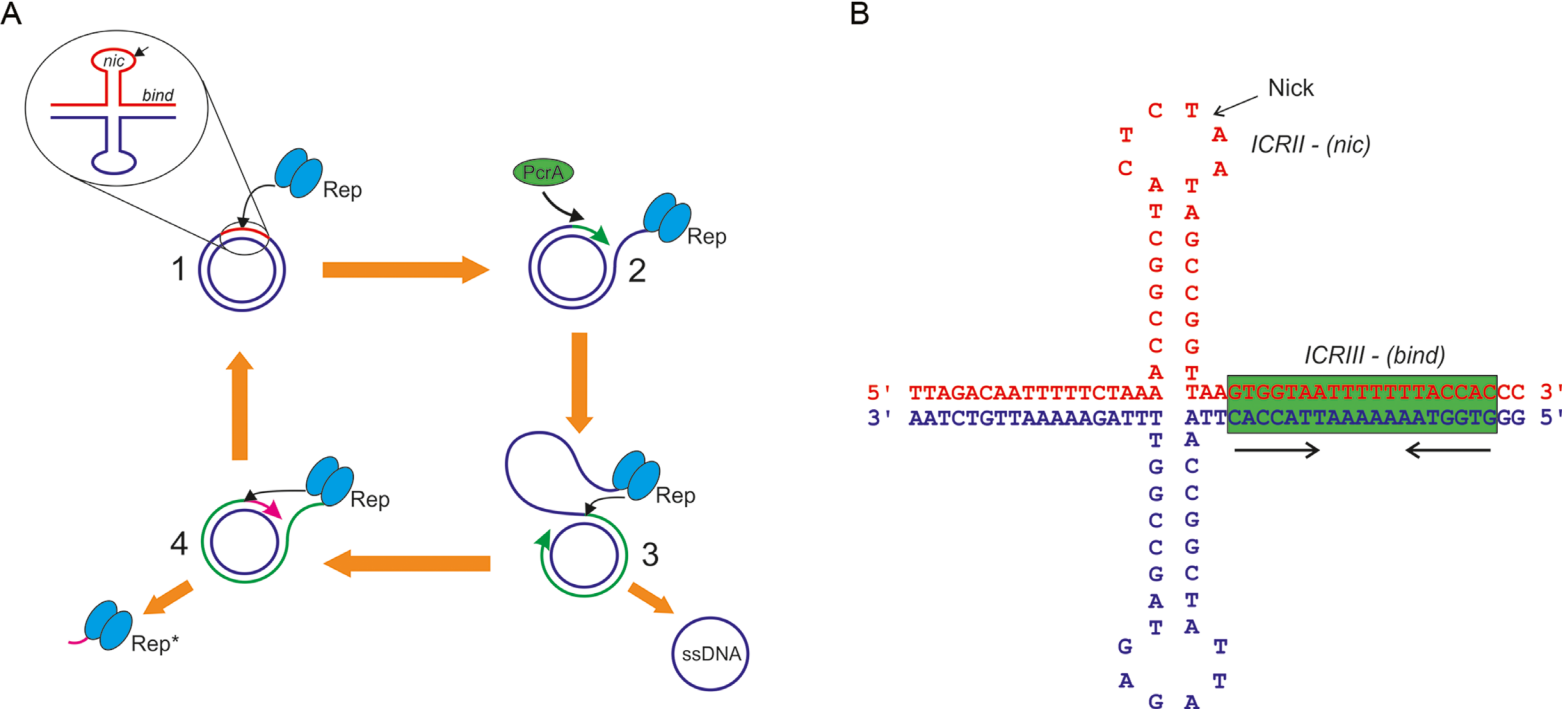
Overview of Rep mediated rolling circle replication. (**A**)(1) Rep protein binds to a hairpin loop at the origin of replication and nicks the (+) strand (red), forming a covalent adduct with the DNA. (2) The Rep protein recruits PcrA helicase to the nick site and following strand separation DNA synthesis commences from the 3′-end of the nick. (3) Synthesis of a new (+) strand (green) displaces the original (+) strand (blue) and on completion the Rep protein nicks the newly synthesized origin and religates the ends of the displaced strand, producing a single stranded product. A new (−) strand is synthesized by the host cell replication machinery. (4) Replication continues 9–11 bases (magenta) beyond the nick site to regenerate the hairpin loop which the Rep protein nicks via one active site while religating the ends of the newly synthesized (+) strand with the other completing synthesis. The 11 base-pair oligonucleotide remains covalently attached to the Rep protein to produce a catalytically inactive Rep* molecule. (**B**) The DNA sequence at the origin of replication of *S. aureus* plasmid pC221. The two inverted repeats are ICRII, which forms a conserved stem–loop structure presenting the nick site at the tip in the (+) strand, and ICRIII, a plasmid-specific repeat which spans the Rep protein binding region and permits discrimination of cognate plasmids by their Rep proteins *in vivo* (highlighted green).

Resistance to antibiotics in pathogenic organisms such as *Staphylococcus aureus* often resides in extra-chromosomal plasmid DNA (1). This is a major concern for human health since the resistance determinants encoded in these plasmids not only render antibiotics ineffective, they are also readily transferred between bacteria, exacerbating the spread of resistance. Such plasmids can be broadly grouped into two classes: the first are larger plasmids of 20 kb or greater that carry multiple resistance markers, while the second are smaller, 5 kb or less, carry a single resistance determinant or may even be cryptic (2). In Gram-positive organisms, the smaller plasmids often replicate via a rolling circle mechanism (Figure 1A) (2,3), a process mediated by a multi-functional replication initiation protein (Rep) encoded on that plasmid.

Replication of pT181 family plasmids (2) is initiated when the dimeric Rep protein makes a sequence specific nick in the (+) strand at the double-stranded origin of replication via one of its active sites, resulting in a covalent adduct to the 5′ side of the nick. The nick site is located in the loop region of a putative stem loop structure, which is followed by a second inverted repeat containing the recognition sequence of the cognate replication initiation protein (Figure 1b). After nicking, the Rep protein assists recruitment of PcrA helicase, which is responsible for unwinding the plasmid during replication. The interaction with the Rep protein not only targets the helicase to its substrate, but also greatly enhances the processivity of the enzyme enabling it to unwind the entire plasmid (4). DNA polymerase III from the host cell commences synthesis of a new (+) strand by extension of the 3′ end, leading to the displacement of the old (+) strand. Once replication of the (+) strand is complete the Rep protein cleaves a second time and religates the two ends of the displaced DNA to produce a single stranded DNA molecule. Synthesis of the (−) strand by host cell factors from a separate single stranded origin completes the replication cycle. While the Rep protein catalyzes the religation of the displaced (+) strand with one active site it simultaneously nicks the newly synthesized strand with the second active site to maintain a covalent link to the DNA. Replication of the (+) strand continues 10–12 nucleotides beyond the nick site, recreating the stem-loop substrate for the Rep protein to perform another cycle of nicking/religation to join the ends of the newly synthesized (+) strand and create an inactivated Rep* protein with the 10–12 nucleotide adduct covalently linked to the catalytic tyrosine. The Rep proteins have also been termed DNA relaxases, since they are capable of nicking and religating negatively supercoiled plasmid DNA carrying a related origin sequence *in vitro*, to form relaxed, covalent-closed products, in a manner similar to that observed for type-I topoisomerases (5).

Sequence comparison of relaxase proteins with those involved in other rolling-circle processes, including phage and virus replication and conjugative DNA transfer (6), has identified two major sub-groups of such proteins. One sub-group includes both the *Rep_1* (PF11446) and *Rep_2* (PF01719) families in the Pfam database (7), and is characterized by a strictly conserved histidine-hydrophobic-histidine (HUH) motif that has been shown to bind a catalytically essential divalent metal ion (8). The molecular structures of several HUH relaxases have been solved (9–12), revealing a highly conserved architecture at the active site, in which a groove suitable for binding single stranded DNA is formed by an α-helix, containing the catalytic tyrosine residue, packing against a β-sheet that also coordinates a divalent metal ion via residues of the HUH motif. The second sub-group is represented by the *Rep_trans* (PF02486) family and includes numerous proteins with the potential to impact human health including: the Rep proteins encoded by antibiotic resistance plasmids of the staphylococcal pT181 family (13); the replication proteins of numerous Gram-negative phage, including CTXΦ, the source of the structural genes for cholera toxin (14); and the relaxase functions of numerous conjugative transposons including Tn916 (15) and ICE*Bs1* (16) that represent an additional pool of mobilisable antibiotic resistance determinants. These proteins share less than 10% sequence identity with the *Rep_1* or *Rep_2* families and lack the conserved HUH motif.

RepD, the replication initiation protein of pT181 family member pC221, has a molecular mass of 37.5 kDa, is dimeric in solution (17,18) and proteolysis experiments show the protein can be divided into three fragments (19,20): (i) a short (3.5 kDa) region at the N-terminus which can be deleted without loss of function *in vitro*; (ii) a central 21 kDa fragment containing the active site tyrosine and residues implicated in the interaction with PcrA helicase (21) and (iii) a 14 kDa C-terminal fragment conferring specific recognition of the replication origin. The C-terminal fragments can be swapped between Rep proteins with a concomitant change in target specificity; however, neither the 21 kDa nor 14 kDa fragments are stable in isolation. Site-directed mutagenesis has identified the active site tyrosine responsible for forming the covalent attachment to DNA along with several other residues critical for activity (17,22,23) that are conserved in the PF02486 motif. These studies also revealed catalysis has an obligate requirement for divalent metal ions; however, since proteins of this family lack the conserved HUH motif the metal coordination site remained undetermined.

No structural information is currently available for any member of the *Rep_trans* family of proteins. Numerous variants of *staphylococcal* Rep proteins of the pT181 family (RepC, D, E, I, J and N) have been subjected to crystal trials, with limited success for RepDC, a hybrid comprised of the 21 kDa fragment of RepD fused to the 14 kDa fragment of RepC (24). In this report we describe the structure determination of the core domain of replication initiator protein of cryptic plasmid pSTK1 (25,26) from *Geobacillus stearothermophilus* (RepSTK1 residues 1–269 (27), hereafter referred to as RepSTK1). This was then used to aid the structure solution of two staphylococcal Rep variants, RepDE and RepDN (containing the 21 kDa fragment of RepD fused to the 14 kDa fragment of RepE or RepN, respectively). The *Geobacillus* and staphylococcal proteins share only 13% sequence identity, yet display remarkable structural conservation. The architecture of the active site and the location of the metal ion required for catalysis are revealed. Additionally, the structures provide clues to how the proteins bind to the origin of replication and suggest a mechanism for PcrA recruitment. They also provide opportunities for the development of novel, potentially broad spectrum antimicrobial agents, since inhibition of such Rep proteins would prevent the replication of numerous plasmids and bacteriophages or the spread of related conjugative transposons containing diverse resistance or pathogenic functions.

## MATERIALS AND METHODS

### Cloning, expression purification of Rep proteins

RepSTK1 was cloned as described previously (27). A full description of the generation of chimeric RepDE and RepDN constructs is provided in the supplementary material, briefly the 21 kDa N-terminal domain of RepD was PCR amplified from plasmid pC221 (using primers F35+/S−) and the 14 kDa C-terminal domains of RepE and RepN were amplified from plasmids pS194 and pCW7 respectively using primer pairs (ES+/EE−) and (S+/Ter−). All primers sequences are listed in supplementary material Supplementary Table S1. The N-terminal domain from RepD was fused with the C-terminal domain of RepE or RepN via SacI sites introduced during the PCR and the resulting chimeric constructs ultimately cloned into pET11a-derived expression vectors via NdeI and BamHI restriction sites.

Following expression, RepDE and RepDN were purified as previously described (19) and both variants were shown to bind and nick DNA with similar activity to RepD (Supplementary Figure S1). RepSTK1 was expressed and purified using a variation of this method (27). Prior to crystallization RepDE and RepDN were dialyzed against 50 mM Tris–HCl pH 7.5, 700 mM KCl, 10% (v/v) ethanediol, and RepSTK1 was dialyzed against 50 mM Tris–HCl pH 7.5, 700 mM KCl. All proteins were concentrated to 5 mg ml^−1^ using Amicon Ultra centrifugal concentrators with a 10 kDa MWCO membrane. All protein concentrations were assessed by absorbance at 280 nm and extinction coefficients calculated from their primary sequences.

### Crystallisation and X-ray Data collection

Crystals of all RepSTK1 were grown as described previously (27). Crystals of RepDE and RepDN were obtained by mixing 500 nl of protein solution with 500 nl 0.5 M ammonium citrate pH 8.5, 15% PEG 8000 or 0.1 M sodium citrate pH 5.5, 2.5 M 1,6-hexane-diol, respectively using an Oryx 6 crystallization robot (Douglas Instruments, Hungerford, UK). Seleno-methionine labelled RepDN required a lower pH of 5.2 for crystal growth. All crystallization experiments were incubated at 294 K and crystals grew within 24 h (RepSTK1) or 5 days (RepDE and RepDN). Crystals were cryoprotected by the addition of 25% (v/v) glycerol (RepSTK1), 25% (v/v) ethanediol (RepDE) or 20% (v/v) ethanediol (RepDN) to the crystallization solution followed by flash-cooling in liquid nitrogen. X-ray diffraction data were collected using multiple beam-lines at Diamond Light Source, ESRF and Daresbury laboratory (Table 1). All data collections were performed at a temperature of 100 K and all data reduction was performed using MOSFLM (28) and either SCALA (29) or AIMLESS (30).

**Table 1.** X-ray data collection and refinement statistics

Protein	RepSTK1	RepSTK1	RepSTK1	RepSTK1	RepDE	RepDN	RepDN
Data set	Native	HgCl_4_	PtCl_4_	MnCl_2_	Native	Native	Selenomethionine
X-ray source	DLS I04–1	DLS I02	DLS I02	DLS I04	DLS I02	Daresbury 14.1	ESRF ID14.2
Space group	*P*2_1_2_1_2_1_	*P*2_1_2_1_2_1_	*P*2_1_2_1_2_1_	*P*2_1_2_1_2_1_	*C*2	*I*2_1_3 (twinned)^d^	*I*4_1_32
Unit cell parameters (Å/°)	*a* = 66.1, *b* = 136.8, *c* = 149.2	*a* = 66.1, *b* = 136.7, *c* = 148.4	*a* = 66.4, *b* = 138.7, *c* = 147.0	*a* = 65.9, *b* = 137.7, *c* = 148.6	*a* = 240.3, *b* = 56.5, *c* = 62.4 *β* = 102.2	*a* = 168.2	*a* = 166.2
Wavelength (Å)	0.9163	1.0073	1.0723	1.8900	0.9796	1.488	0.9794
Resolution (Å)	40.2–2.30 (2.36–2.30)	68.3–2.66 (2.73–2.66)	66.4–3.49 (3.58–3.49)	65.4–4.0 (4.15 -4.0)	49.9–2.9 (3.06–2.9)	39.7–3.0 (3.07–3.0)	67.9–3.0 (3.16–3.0)
Unique reflections	60,795 (4,408)	39,338 (2,848)	17,928 (1,261)	11,664 (862)	18,387 (2,676)	15,902 (2,299)	8,164 (1,153)
Completeness (%)	99.7 (99.0)	99.9 (99.9)	99.9 (100)	99.9 (99.9)	99.6 (100)	99.9 (100)	100 (100)
Multiplicity	7.0 (7.1)	6.6 (6.8)	6.5 (6.7)	14.1 (14.4)	3.5 (3.6)	10.3 (4.7)	40.1 (38.4)
<*I*/*σI*>	16.9 (2.2)	19.8 (2.8)	20.7 (3.4)	19.4 (4.4)	9.3 (1.5)	23.7 (2.3)	34.7 (2.9)
*R*_merge_ (%)^a^	5.3 (86.5)	5.1 (63.5)	5.7 (52.8)	11.1 (64.2)	12.9 (59.4)	7.9 (56)	15.5 (139.6)
*R*_pim_ (%)^b^	2.3 (36.8)	3.2 (39.5)	2.9 (23.8)	3.2 (18.0)	8.2 (36.8)	2.5 (28.5)	2.7 (22.9)
Anomalous completeness (%)		99.3 (99.5)	99.4 (99.5)	99.9 (99.9)			100 (100)
Anomalous multiplicity		3.3 (3.3)	3.4 (3.4)	7.7 (7.7)			21.7 (20.0)
Anomalous mid-slope		1.304	1.248	1.221			1.664
**Refinement**							
*R*_work_/*R*_free_^c^ (%)	19.3/ 23.5				21.5/25.9	24.1/29.7	
Model							
Protein atoms	8720				4600	4596	
Water molecules	268				0	0	
R.M.S.D. bond lengths (Å)	0.014				0.011	0.011	
R.M.S.D. Bond Angles (°)	1.54				1.49	1.58	
Ramachandran Favored/Outliers (%)	97.6/0				95.7/1	95.5/0	

Numbers in parenthesis refer to highest resolution shell.

^a^*R*_merge_ = Σ_*hkl*_Σ_*i*_|*I_i_*(*hkl*) − <*I_i_*(*hkl*)>|/Σ_*hkl*_Σ*I_i_*(*hkl*), where *I_i_*(*hkl*) is the intensity of reflection *hkl* and Σ_*i*_ is the sum over all i measurements of reflection *hkl*.

^b^*R*_pim_ = Σ_*hkl*_(1/*N* − 1)^1/2^ Σ_*i*_|*I_i_*(*hkl*) − <*I*(*hkl*)>|/ Σ_*hkl*_Σ*_i_I_i_*(*hkl*) where *I* is the integrated intensity of a given reflection and <*I*> is the mean intensity of multiple corresponding, symmetry related reflections and *N* is the multiplicity of a given reflection.

^c^*R*_work_ = Σ_*hkl*_∥*F*_obs_| − *F*_calc_∥/Σ_*hkl*_|*F*_obs_| where *F*_obs_ and *F*_calc_ are the observed and calculated structure factors respectively. *R*_free_ is calculated in the same manner, but using a random subset (5%) of reflections that are excluded from refinement.

^d^The crystals of RepDN were all of the same space group (*I*2_1_3 with merohedral twinning giving an apparent symmetry of *I*4_1_32). Selenomethionine data processed in the higher apparent symmetry ignoring twinning produced more interpretable electron density maps, but the final model was refined in the true space group taking account of the twinning.

### Structure solution and refinement

Initial phase estimates for RepSTK1 were obtained from a MIRAS phasing experiment with crystals soaked in 1 mM HgCl_2_ or K_2_PtCl_4_ for 10–20 min prior to flash-cooling. Diffraction data were collected at the *k*-edge of each heavy atom and their positions identified, refined and phases calculated using the autoSHARP pipeline (31). The resultant electron density maps were of sufficient quality to allow automated chain tracing using BUCCANEER (32) to produce an initial model, which was refined using REFMAC5 (33). Model phases were combined with experimental phases to improve the quality of the electron density map. Further rounds of manual rebuilding in Coot (34) and refinement with REFMAC5 completed the model. To locate the metal ion in the active site crystals were soaked in reservoir solution containing 10 mM MnCl_2_ prior to cryo-cooling and diffraction data collected at the Mn *K*-edge. Anomalous difference maps were calculated by combining model phases with manganese anomalous differences using FFT (35).

Attempts to generate phase estimates for RepDE or RepDN by molecular replacement using RepSTK1 as a search model were unsuccessful, however, SAD phasing was possible using selenomethionine labelled RepDN. The twinned, selenomethionine-labelled data were processed in the higher apparent symmetry space group *I*4_1_32, resulting from a combination of the true space-group and the twinning operator. Selenium atoms were located and phases calculated using autoSHARP, producing electron density maps in which some secondary structural motifs were visible. Ideal alpha-helices and beta-sheets were manually docked into electron density using Coot to produce an initial model. Fragments of the RepSTK1 structure were superposed onto the partial model of RepDN and used as a guide to aid further building of the RepDN structure. Iterative rounds of refinement (REFMAC), phase combination and manual rebuilding, using RepSTK1 as a guide, were continued until no further electron density could be interpreted, to produce a model for much of the catalytic domain of RepDN.

The model of the catalytic domain of RepDN was used as a search model to generate phase estimates for RepDE by molecular replacement using Phaser (36). The resulting electron density maps showed clear density for the missing regions of the catalytic domain, but also additionally for the DNA binding domain. Automatic chain tracing with BUCCANEER completed the model, followed iteratively by cycles of refinement in REFMAC and manual rebuilding in Coot. One monomer of RepDE was then used as a search model to calculate phases for RepDN in the true space group (*I*2_1_3). Prime-and-Switch density modified maps were calculated using RESOLVE (37) and the search model was manually fitted to these maps using Coot. The resulting model was subject to rounds of refinement using REFMAC and rebuilding as described for RepDE. During the refinement steps structural restraints were calculated from the RepDE model using PROSMART (38) and applied to the RepDN model. Any models were made by manually docking molecules in Coot with no further refinement.

## RESULTS

### Structures of RepSTK1, RepDE and RepDN

**Figure 2.**
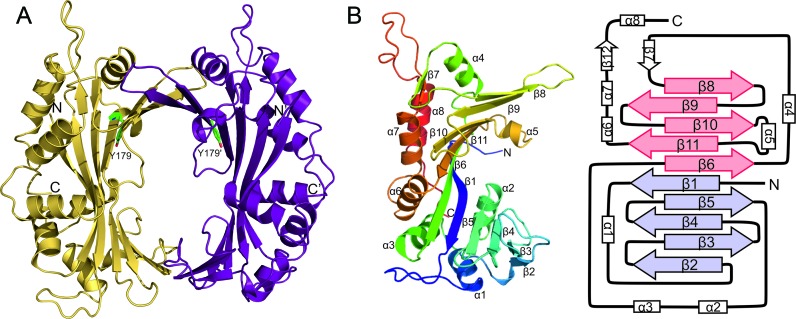
X-ray crystal structure of RepSTK1 from *Geobacillus stearothermophilus*. (**A**) Cartoon representation of RepSTK1 with the catalytic tyrosine residues displayed as green sticks. (**B**) A monomer of RepSTK1 colored blue at the N-terminus through to red at the C-terminus with the location of each strand and helix identified. The structure is also shown schematically with the two 5-strand modules found in the major β-sheet coloured magenta and cyan to highlight the pseudo-symmetry within this part of the structure.

RepSTK1 formed crystals belonging to space-group *P*2_1_2_1_2_1_ with four molecules per asymmetric unit, arranged as two dimers (27). The structure was determined to a resolution of 2.3 Å by MIRAS phasing using mercury chloride and potassium chloroplatinate derivatives (Table 1). Each subunit is crescent shaped (Figure 2A), with a concave inner surface formed by a 10-stranded, antiparallel β-sheet the outer surface of which is decorated with eight α-helices. Numbering the strands of the sheet sequentially according to their order in the primary sequence (Figure 2B) reveals an unusual topology. The strand closest to the N-terminus of the protein lies at the centre of the sheet and the lower half of the crescent contains strands 2–5 with strand 5 packing against strand 1. Strand 6 abuts the opposite side of strand 1 with strands 8–11 forming the remainder of the sheet. The conserved residues of the *Rep_trans* motif are located in adjacent strands 6, 9, 10 and 11, suggesting this is the catalytic centre of the protein. This topology produces a sheet consisting of two 5-strand modules related by 2-fold pseudo-symmetry (Figure 2B). The subunits interact via interfaces at either end of the crescent to form a ring where the diameter of the central cavity is 20 Å. One interface, formed by strand 8 from each subunit, is highly extended burying 2234 Å^2^ of the protein surface and stabilized by 23 hydrogen-bonds and 4 salt bridges. The second is formed between inter-strand loops at the opposite end of the sheet and is much smaller burying 676 Å^2^ and stabilized by a single hydrogen bond (Supplementary Figure S2) and two salt bridges.

Constructs of RepDE and RepDN spanning residues 35–314 were used for crystallisation studies, since the first 34 amino acids are predicted to be disordered and had previously been shown to be dispensable for protein function (19,20). Solving the structures of the staphylococcal proteins presented a number of challenges. Crystals of RepDN belong to space group *I*2_1_3, but the data were near perfectly twinned (refined twin fraction of 49.8%) giving an apparent space group of *I*4_1_32. The twinning severely hampered experimental phasing; however, it was possible to produce some partially interpretable electron density maps using SIRAS data collected from seleno-methionine labelled crystals. RepDE crystallized in space group *C*2, but all attempts to generate experimental phases were unsuccessful. A partial model of RepDN, built into the twinned SIRAS map, produced a solution in molecular replacement phasing experiments against the RepDE data, but this model was too incomplete to allow further model building or refinement. Molecular replacement with the RepSTK1 coordinates was also unsuccessful, but manual docking of fragments of the RepSTK1 model into the RepDN density allowed further interpretation of the RepDN electron density maps. Molecular replacement with the more complete RepDN model and RepDE data resulted in electron density maps in which an additional domain was visible. Multiple rounds of rebuilding and refinement completed the model of RepDE, which in turn was used to complete the model of RepDN. Table 1 also shows the phasing and refinement statistics for RepDE and RepDN.

**Figure 3.**
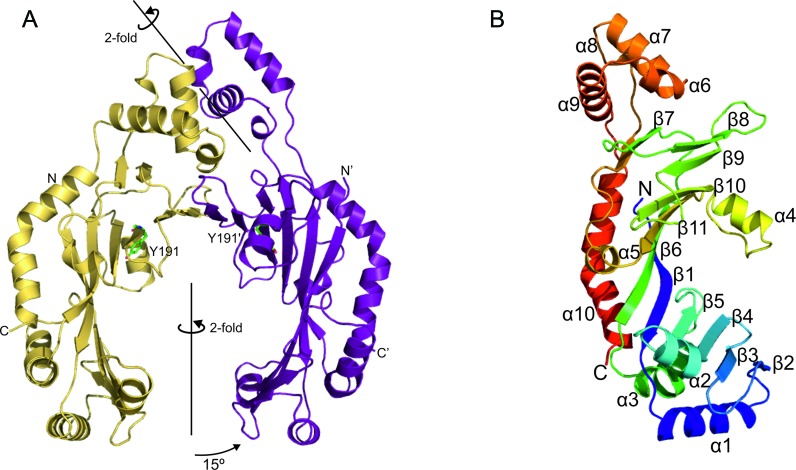
X-ray crystal structure of RepDE from *Staphylococcus aureus*. (**A**) Cartoon representation of RepDE with the location of the catalytic tyrosine residues shown as green sticks. The 2-fold, non-crystallographic symmetry axes of both the catalytic and DNA binding domains (DBD) are indicated, highlighting the tilted conformation of the DBD with respect the catalytic domain. The 15° rotation of the catalytic domain relative to its position in RepSTK1 is also shown. RepDN is not shown, but is structurally very similar to RepDE. (**B)** Monomer of RepDE with rainbow colouring from blue at the N-terminus to red at the C-terminus with each strand and helix identified.

Crystals of both RepDE and RepDN contain two molecules per asymmetric unit as a non-crystallographic dimer and the final structural model spans residues 39–308 for all subunits. The catalytic residues of RepDE are found in the crescent shaped domain, within a 10-stranded β-sheet, the outer surface of which is decorated with 7 α-helices (Figure 3a). In this protein the catalytic domains do not form a closed ring, but rather a ‘horseshoe’ where they are rotated by 15° relative to their position in RepSTK1. The relative orientation of the catalytic domains in RepDN is intermediate between those observed in the other two proteins. They still form a ‘horseshoe’, but are rotated 5° towards each other relative to the position in RepDE, reducing the distance between protein chains at the open end of the ‘horseshoe’ by 5 Å (Supplementary Figure S3). The arrangement of the strands within the β-sheet of the catalytic domain is identical to that in RepSTK1, but there is no structural conservation between the α-helices (Figures 2B and 3B) except helix α4 (α5 in RepSTK1), which lies close to the inner face of the β-sheet and contains the semi-conserved residues Q184 (RepSTK1) and E196 (RepDE/RepDN) of the *Rep_trans* motif.

RepDE and RepDN also contain an additional all-helical domain (helices α6–α9, residues 237–280) previously identified to contain the residues responsible for recognition of the ICRIII sequence at the origin of replication, conferring specificity between the protein and its plasmid substrate (39). This domain also contributes further to the interface between the two polypeptide chains in the dimer. These domains form a dimer with a non-crystallographic symmetry axis that is tilted 128° relative to the non-crystallographic axis of the catalytic domains (Figure 3a), and together form the DNA binding domain (DBD). The tilting of the DBD forms an interface with the catalytic domain that is stabilized by six hydrogen-bonds, multiple hydrophobic contacts and buries 1775 Å^2^ of the solvent accessible area of the protein.

### Structure comparisons between RepSTK1, RepDE and other proteins

Comparison of the catalytic domain of RepDE with RepSTK1 reveals the conformation of the β-sheet is highly conserved, whereas the positions of α-helices, with the exception of helix α4/5 and the C-terminal helix show no similarities at all. Structural comparison of RepSTK1 and the catalytic domain of RepDE to other proteins using the DALI server (40) reveals no homologues, but similarity (DALI *Z*-score of 5.4) with the extended β-sheet in TATA binding protein (TBP) (41,42; pdb 1d3u). This is strictly limited to the β-sheets, which can be superposed with an r.m.s.d. of 3.5 Å between C_α_ positions of the 68 residues that comprise the β-sheets of each protein, with no superposition possible for the helices (Supplementary Figure S4a). Each subunit in the DBD superposes with the DNA binding domains of various proteins including RNA polymerase sigma factors (43; pdb 4g6d, DALI *Z*-score 2.5) and transcription factors (44; pdb 2ivm, DALI *Z*-score 2.4), with r.m.s.d. values in C_α_ positions (calculated using all 46 residues in the DBD) of 2.0 and 2.6 Å, respectively (Supplementary Figure S4b).

### The active sites of RepDE and RepSTK1

**Figure 4.**
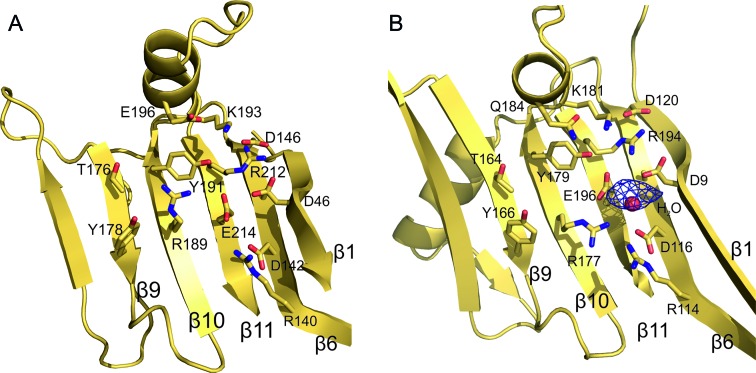
RepDE and RepSTK1 share a common active site architecture. (**A**) The active site of RepDE. The structure reveals that the conserved residues of the *Rep_trans* family cluster around the catalytic tyrosine residue to form the active site. (**B**) The catalytic centre of RepSTK1 shows a remarkable conservation of structure between the two proteins with all of the side chains adopting identical conformations except R189 (R177 in RepSTK1). The electron density shown is anomalous difference density calculated from diffraction data collected at the Mn *K*-edge from a RepSTK1 crystal soaked in 10 mM MnCl_2_, revealing the position of the metal ion in the active site. The structure shown was crystallized from a metal free solution where a water molecule (red sphere) occupies the metal binding site.

The Rep proteins possess two related catalytic activities, a type-I topoisomerase-like cleavage activity in which they form a nick in the (+) strand at the origin of replication to initiate DNA synthesis, and a religation activity to join the ends of the parental strand that is displaced during replication. Tyrosine 191 acts as the nucleophile during the nicking/religation reactions in staphylococcal Rep proteins (17, Y179 is the nucleophile in RepSTK1; *G. stearothermophilus* residue numbers will be shown in brackets from this point on) and is located on strand 10 pointing into the cavity formed between the two large β-sheets (Figures 2A and 4A). Sequence alignment of proteins containing the *Rep_trans* motif has revealed a number of conserved residues adjacent to the active site tyrosine, which may also be involved in catalysis (Supplementary Figure S5) These include R189 (R177), K193 (K181), E196 (Q184), T176 (T164), Y178 (Y166) and G180 (G168). Mapping these residues onto the structure reveals all but E196 (Q184) lie on the inner face of the major β-sheet in strands 9 and 10 adjacent to the catalytic tyrosine, with their side chains protruding into the central cavity (Figure 4) Glutamate 196 resides on helix α4 (Q184 is found on helix α5) close to the catalytic tyrosine. The side chains of both amino acids point towards the central cavity of the catalytic domain. The proximity of this cluster of residues to Y191 is suggestive of a role in catalysis and alanine scanning mutagenesis of these residues in RepD yields proteins with a reduced ability to nick and/or religate DNA (17,22). The most extreme loss of activity is displayed by mutants R189A (45) and K193A, which both exhibit an impaired nicking activity and a significantly reduced ability to perform the religation reaction. The lack of identity between E196 and (Q184) is suggestive of a subtle difference between the catalytic mechanisms of the two proteins; however, the mutation E196Q has minimal effect on enzymatic turnover [unpublished observation]. Sequence analysis also reveals strict conservation of R140 (R114), D142 (D116), A144 (A118), D146 (D120), R212 (R194) and E214 (E196) in the *Rep_trans* motif. These are also found on the inward-facing surface of strands 11 and 6 close to Y191 (Figure 4) again suggesting a possible functional role for these residues as well. Comparison of the active sites of RepDE and RepSTK1 show that they are remarkably similar and can be superposed with an r.m.s.d. of 0.4 Å between the C_α_ positions of the catalytic residues. The similarity extends further with the side chains of residues in each active site adopting identical conformations, with the exception of R189 (R177).

The topoisomerase activity of the Rep proteins has an absolute requirement for the presence of divalent metal ions. The highest peak (0.05 electrons/Å^3^, 5.3*σ*) in anomalous difference maps calculated using data collected from RepSTK1 crystals soaked in manganese solutions is found near the catalytic tyrosine identifying the position of the metal ion (Figure 4B). The metal lies towards one side of the putative active site and is co-ordinated by three amino acids: E214 (E196), D142 (D116) and D46 (D9). Mutation of each of the metal coordinating residues results in a significant reduction or complete loss of topoisomerase activity *in vitro* (Supplementary Figure S6). The model of RepSTK1, crystallized in the absence of divalent metal ions, contains a water molecule in the metal binding site. A structural model with metal bound has not been produced due to the low resolution of the diffraction data (Table 1).

### DNA binding and PcrA interaction interfaces of the Rep proteins

**Figure 5.**
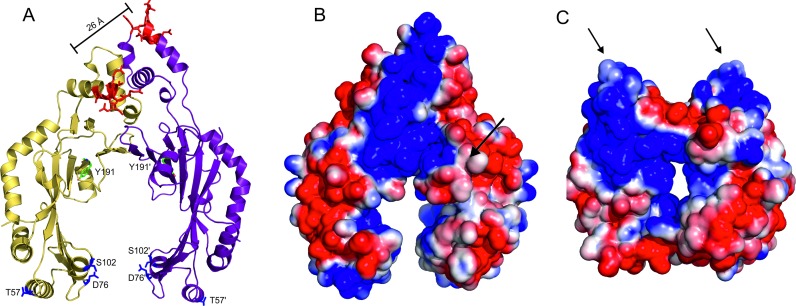
The location of DNA and PcrA binding interfaces. (**A)** The structure of RepDE with the DNA binding interface shown as red sticks, and the location of *pcrA3* suppressor mutations shown as blue sticks. The catalytic tyrosine is also indicated. (**B)** Electrostatic surface representation of RepDE with the location of the protease K sensitive loop indicated by an arrow. (**C**) Electrostatic surface representation of RepSTK1, with the two basic loops which take the place of the DBD indicated with arrows.

Each subunit of the DBD is formed by four α-helices, with helices α8 and α9 (residues 258–278) adopting a conformation similar to that observed for a helix–turn–helix (HTH) DNA recognition motif (46). The amino-acids responsible for DNA binding are well characterized for the pT181 family of Rep proteins, spanning residues 265–270 (39) at the N-terminus of the second helix in the HTH-like motif. The two DNA recognition helices are separated by 26 Å across the 2-fold non-crystallographic symmetry axis on one face of the DBD dimer (Figure 5a). Calculation of surface electrostatic charge shows that this region of the protein is highly basic, as expected for a DNA binding interface. The positive charges are, however, not limited to the DNA recognition motifs, but extend into the catalytic domain towards the active site (Figure 5B). The interior cavity of the catalytic domains is also positively charged, when calculated with a divalent metal ion modelled into the active site. The DNA binding residues of RepSTK1 have not been identified, but two extended loops protrude from the surface of the protein (the top of the protein as shown in Figures 2A and 5C) replacing the separate DBD found in the staphylococcal proteins. These loops form a basic channel with suitable dimensions to accommodate dsDNA, suggesting this could be a site of DNA interaction. The positively charged surface of RepSTK1 also extends from the putative DNA binding region towards the catalytic residues. The staphylococcal and *Geobacillus* Rep proteins have both been found to stimulate the activity of DNA helicase PcrA (4 and unpublished observations), which is an obligate requirement for replication of the pT181 family of plasmids (47). Previous genetic studies with RepC to identify suppressors of the *pcrA3* mutation had identified residues D57, D76 and S102 (*S. aureus* numbering) as relevant to this interaction (21). Mapping these residues onto the RepDE structure identifies a potential interaction interface for PcrA helicase, at the open end of the catalytic domain (Figure 5A). It should be noted that although RepSTK1 also interacts with PcrA during replication there is no apparent sequence conservation at the putative helicase binding site.

## DISCUSSION

Despite low overall sequence identity, there is a high degree of structural conservation between the catalytic domains of the Rep proteins from these *Geobacillus stearothermophilus* and *Staphylococcus aureus* plasmids. Structure-based sequence alignment reveals the conserved residues are almost exclusively located in the β-strands containing the *Rep_trans* motifs, and it is likely the crescent-shaped catalytic domain represents the canonical fold for the *Rep_trans* protein family. The most strictly conserved residues in the *Phage_Cri* family of replication proteins are arranged in motifs similar to those observed for the *Rep_trans* family (Supplementary Figure S5), suggesting a comparable arrangement of residues at the catalytic centre could be achieved if members of the *Phage_Cri* family also adopt a similar crescent-shaped fold. The topology of the β-sheet is uncommon, but not unique in the PDB, with TBP also containing an extended, highly curved sheet where the strands show the same connectivity and internal symmetry. In TBP, this symmetry is reflected in the primary sequence and has been proposed to have arisen from a gene duplication event (48), however, there is no evidence of symmetry in the primary sequence of either Rep protein.

The differing quaternary structures of the catalytic domains of the Rep proteins may represent functionally relevant conformations, since the active site lies on the inner surface of the β-sheet and the diameter of the central cavity of RepSTK1 would severely hinder access of the dsDNA substrate. Any steric hindrance to DNA access would be greatly reduced by the catalytic domains swinging apart to open the ring and adopting the ‘horseshoe’ conformation observed for both staphylococcal proteins. The different conformations adopted by RepDE and RepDN demonstrate the catalytic domains can move relative to one another to modify the size of the cavity containing the active sites. Enabling RepSTK1 to undergo such a conformational change would require disruption of one of the interfaces stabilising the ring, with the smaller of the two interfaces (Supplementary Figure S2b) needing significantly less energy to disrupt. Since *Geobacillus stearothermophilus* typically grows at 65°C the kinetic energy of the protein at this temperature could be sufficient to enable ring opening, and *in vitro* nicking assays with RepSTK1 show significantly reduced DNA cleavage at temperatures <65°C. The remainder of the discussion will focus primarily the staphylococcal Rep proteins, since all the biochemical data we will discuss in relation to the structures were obtained using members of the pT181 family of replication initiation proteins.

The fold of the catalytic domain is significantly different from that observed for the HUH relaxases (9–12), which consist of an α-helix containing the catalytic tyrosine residue packing against a β-sheet containing the metal coordinating histidine residues. Both of which are adjacent to a narrow groove capable of binding single stranded DNA (49). In contrast, all of the residues necessary for catalysis in the *Rep_trans* family of relaxases lie on the inner face of the β-sheet in the catalytic domain, with those co-ordinating the metal ion each on a separate strand. Despite the differences in protein architecture there are some similarities between the active sites of these distinct families. The side chains of the catalytic tyrosine residues adopt a similar orientation with respect to the metal ions and are separated from them by ∼5 Å (Supplementary Figure S7a). Such similarities might be expected since relaxases from both families catalyze the same nicking reaction and may represent a case of convergent evolution. The arrangement of catalytic tyrosine and metal binding site formed from three carboxylate groups is also similar to that observed in type II topoisomerases. The active site of topoisomerase II, however, is assembled by bringing together a tyrosine from the gyrase domain of the topoisomerase A subunit with the metal ion coordinating residues from the TOPRIM domain in the topoisomerase B subunit in a multi-protein complex (50) (Supplementary Figure S7b). The similarities are strictly limited to the metal binding site and tyrosine residue, since, for example, no equivalent residues to the basic amino acids R189 or K193 that are critical for religation can be found in HUH family relaxases or type II topoisomerases.

The DBD of staphylococcal Rep proteins confers substrate specificity by targeting the palindromic ICRIII region in the double-stranded origin of replication of the parent plasmid. The sequence recognised spans 19 base-pairs or two turns of dsDNA (19). The location of the DNA binding residues is reminiscent of a recognition helix in a HTH motif (46); however helices 8 and 9 do not form a true HTH since their relative orientation differs from that typically observed for this structural motif. The DNA binding residues of the DBD are separated by about 26 Å across the two-fold axis and a B-form DNA model of ICRIII can readily be docked onto the DBD such that the DNA binding residues interact with two consecutive turns of the major groove (Figure 5a). The recognition sequence of the Rep proteins is centred 23 base-pairs downstream of the nick site (Figure 1b) and the separation of the DNA recognition helices and active site is appropriate for the protein to interact simultaneously with the ICRIII target sequence and the nick site if the latter is extruded as a stem–loop structure. A similar separation of the DNA binding interface and catalytic centre has been observed for the HUH relaxases from adeno-associated virus (51) and porcine circovirus (52), both of which bind to recognition sequences adjacent to a stem-loop that contains the nick site. The electrostatic charge distribution across the surface of the protein shows the putative DNA binding interface on the DBD is highly basic, and the positively charged surface extends beyond the binding interface towards the active site of the protein, suggesting the interaction interface with DNA extends beyond the recognition helices. A loop adjacent to the basic patch is susceptible to cleavage by proteinase K (between residues N206 and S207) (20) (Figure 5b), in the absence of DNA, but is protected when a covalent adduct is formed following DNA nicking, suggesting DNA interacts with this region of the protein.

The orientation of the DBD, with its two-fold axis tilted relative to the two-fold axis of the catalytic domain, is observed in crystals of both RepDE and RepDN. These have different crystal symmetries and lattice contacts suggesting the tilt is probably not an artefact of crystal packing. The interface between the catalytic domains and DNA-binding domain stabilising the ‘tilted’ conformation has similar characteristics to interfaces that stabilize transient protein-protein interactions (53), suggesting the domains could move relative to one another. The tilting could help orient the active site towards the nick site once the protein has bound to the target sequence downstream of the stem-loop.

**Figure 6.**
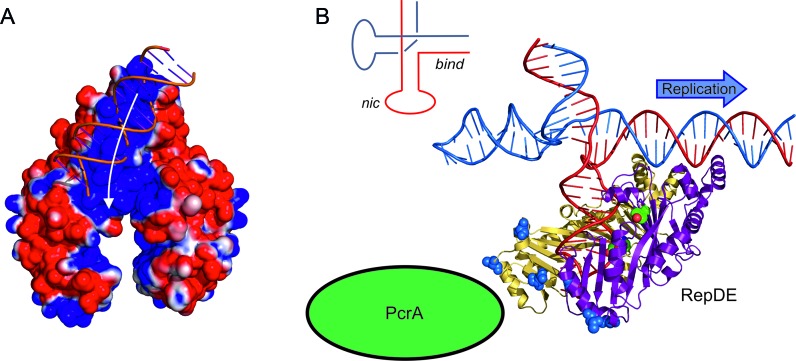
Recognition of the Origin of replication by RepDE. (**A)** Electrostatic surface of RepDE with a B-DNA duplex docked onto the DNA binding region, the white arrow shows the possible path the DNA would need to take in order to access the active site. (**B**) The origin of replication modelled as a Holliday junction, where the coaxial arrangement of DNA duplexes allows the DNA binding domain of the protein to interact with the target sequence while positioning the nick site close to the catalytic tyrosine (green spheres). This orientation of the Rep protein also presents the PcrA interaction interface (blue spheres) immediately upstream of the nick site presumably aiding recruitment of the helicase (green) to the DNA. The schematic of the origin of replication inset shows the conformation the DNA strands in the Holliday junction.

Mapping residues responsible for catalysis, DNA binding and PcrA stimulation onto the structure of RepDE allows us propose models for how the staphylococcal Rep proteins could initiate replication, recruit PcrA and enhance the processivity of the helicase. The origin of replication in pT181 family plasmids contains two inverted repeats: the first is extruded as a stem loop and contains the nick site, while the second contains the target sequence recognised by the Rep protein (Figure 1). The Rep protein binds to its target sequence via the DBD and the tilt of the catalytic domain relative to the DBD creates a continuous positively charged surface linking the DNA recognition helices with the active site. Ideal B-form DNA can be docked onto the positively charged surface of the DBD (Figure 6a), however, further interaction with the basic surface that enters the catalytic domain requires the DNA to bend or adopt a more complex tertiary structure, for example, a Holliday-junction. Assuming the extruded hairpins adopt a Holliday-junction-like conformation, with coaxially stacked duplexes (54), a model of the double strand origin can be constructed from ideal B-form DNA, a Holliday junction (for example, pbd 1dcw) and two 6 base-pair loops (pdb 1mtw), enabling us to model how the RepDE might interact with this substrate. In such a model the centre of the ICRIII binding site and nick site are separated by 40 Å, similar to the distance of 38 Å between the hydroxyl group of Y191 and the centre of the DBD. Holliday junctions are capable of adopting multiple conformations and a model based upon the conformation observed in complex with T7 Endonuclease I (55) suggests a substrate where the DBD could bind at ICRIII while ICRII occupies the active site (Figure 6B). In this conformation, the DNA interacts with the full length of the basic interface linking the DBD with the active site without the need for DNA bending and the scissile phosphate lies near Y191. Additionally, the hairpin loop formed by ICRII lies next to the proteinase K sensitive loop, which could, like the covalent adduct formed after DNA nicking, protect it from proteolysis. The model suggests that loss of the hairpin structure would produce a substrate in which the nick site is incorrectly presented to the active site thus inhibiting DNA cleavage.

**Figure 7.**
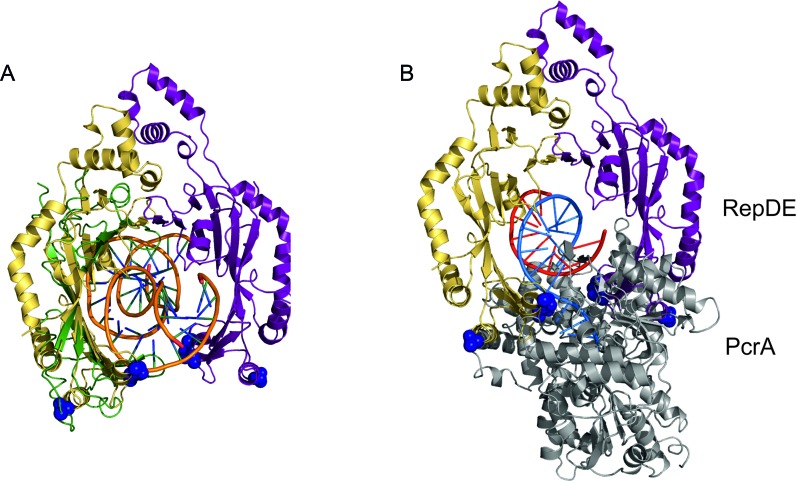
RepDE binds PcrA helicase to form a processive complex. (**A**) Model of DNA encircled by the RepDE catalytic domains (yellow and purple) after DNA nicking based on the binding of DNA by TATA-binding protein (green). The inner face of the catalytic domain is basic in character and could interact non-specifically with the DNA backbone. With the DNA bound in this manner the interaction of PcrA with the supposed contact residues (blue spheres) would ‘lock’ the helicase onto the substrate. (**B**) Positioning the duplex DNA present in the PcrA-DNA complex (pdb 3pjr) suggests how the Rep protein might interact with PcrA (gray). Two wide grooves, between domains 2A and 1/2B on the helicase, could represent the Rep binding interface. This interaction creates a ternary complex in which the two proteins completely encircle the DNA, with the Rep protein stabilising the complex between the helicase and its substrate.

After nicking at the origin replication enters a processive phase as the Rep protein recruits PcrA helicase to unwind the plasmid and permit DNA synthesis. Translation of the Rep protein along the DNA in conjunction with PcrA then maintains processivity of the helicase (4,45). Such remodelling of the Rep-DNA complex to permit stimulation of the helicase and release of the ICRIII binding sequence (Figure 1A) from the DBD may be driven by the release of superhelical tension on cleavage. A likely docking site for the duplex DNA would be the basic inner surface of the catalytic domains, since the nicked DNA is covalently tethered to this region. Covalent attachment of the Rep protein to the 5′ side of the nick site in the (+) strand creates an asymmetric substrate for interaction with PcrA that contains a short stretch of single stranded DNA in the (−) strand. Presentation of single stranded DNA to the helicase or the asymmetry of the nicked DNA substrate when Rep attaches are two possible causes of the directional loading of PcrA onto such nicked DNA (56). A model of such a complex may be produced by superposition of the β-sheets in RepDE and the TBP–DNA complex (42), where the DNA interacts with the inner surface of an almost identical β-sheet. This superposition places a DNA duplex between the two catalytic subunits of RepDE such that it would be almost completely encircled (Figure 7a), with RepDE residues identified as important to the interaction with PcrA exposed to solvent. With the DNA in this position, interaction with PcrA would effectively lock the helicase onto the substrate next to the exposed (−) strand, enhancing the processivity of the unwinding reaction. The structure of PcrA in complex with DNA has been solved (pdb 3pjr), and in this structure the DNA has both double and single stranded regions. Assuming the duplex DNA lies in a similar position to that observed in 3pjr, superposition of the duplex into the DNA in the Rep-DNA model shown in Figure 7a leads to a possible approximate model for the RepDE-PcrA-DNA ternary complex (Figure 7B). In this model the two basic PcrA interaction interfaces at the open end of the RepDE catalytic horseshoe occupy two acidic grooves on the surface of PcrA (between domains 2A and 1B/2B). Opening of the ring structure of RepSTK1 exposes two basic patches positioned to dock into the acidic grooves on PcrA, indicating that the overall charge distribution of the PcrA binding site is conserved between the two homologues even in the absence of any clear sequence conservation. The positioning of either Rep protein would not impede any conformational changes in PcrA during DNA unwinding. As well as enhancing processivity, encircling the DNA would also provide a mechanism for the Rep protein to monitor the displaced (+) strand for the next encounter with the origin of replication to ensure correct termination of DNA synthesis.

## ACCESSION NUMBERS

Coordinates and structure factors have been deposited in the protein data bank with accession codes 4cij (RepSTK1), 4cwc (RepDE) and 4cwe (RepDN).

## Supplementary Material

SUPPLEMENTARY DATA
