# Supplementary material for: Structures of replication initiation proteins from staphylococcal antibiotic resistance plasmids reveal protein asymmetry and flexibility are necessary for replication
Source: Nucleic Acids Res. 2016 Jan 20;44(5):2417–28. doi: 10.1093/nar/gkv1539 (PMC4797284; doi:10.1093/nar/gkv1539)
Supplement: SUPPLEMENTARY DATA [file supp_44_5_2417__index.html]

Structures of replication initiation proteins from staphylococcal antibiotic resistance plasmids reveal protein asymmetry and flexibility are necessary for replication — Structures of replication initiation proteins from staphylococcal antibiotic resistance plasmids reveal protein asymmetry and flexibility are necessary for replication — SUPPLEMENTARY DATA 

# Structures of replication initiation proteins from staphylococcal antibiotic resistance plasmids reveal protein asymmetry and flexibility are necessary for replication

## SUPPLEMENTARY DATA

- SUPPLEMENTARY DATA
