# Supplementary material for: Structures of replication initiation proteins from staphylococcal antibiotic resistance plasmids reveal protein asymmetry and flexibility are necessary for replication
Source: Nucleic Acids Res. 2016 Jan 20;44(5):2417–28. doi: 10.1093/nar/gkv1539 (PMC4797284; doi:10.1093/nar/gkv1539)
Supplement: SUPPLEMENTARY DATA [file supp_gkv1539_nar-03266-h-2015-File010.pdf]

Supplementary Material : Structures of Replication Initiation Proteins from staphylococcal antibiotic resistance plasmids reveal asymmetry and flexibility are necessary for replication.

Stephen B. Carr, Simon E.V. Phillips, Christopher D. Thomas

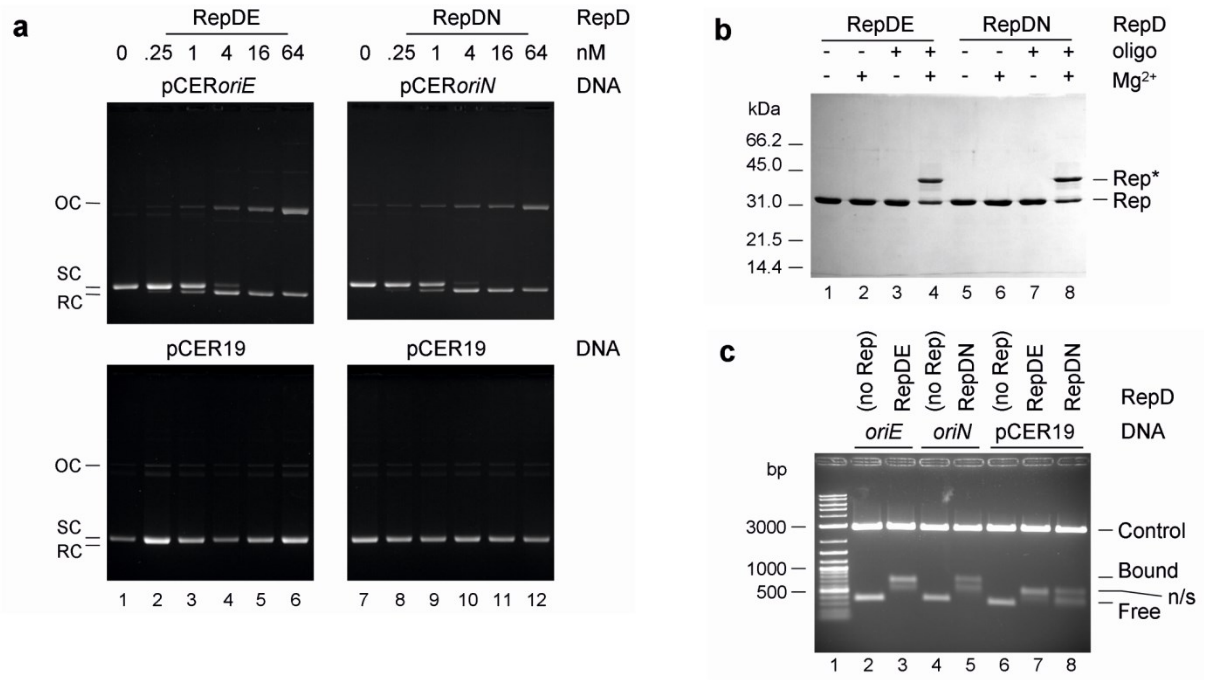

Supplementary Figure S1 - Catalytic activities of RepDE and RepDN

a RepDE and RepDN fusion proteins display sequence-specific topoisomerase activity against negatively-supercoiled substrates containing the replication origins of pS194 (pCER*oriE*), pCW7 (pCER*oriN*) with no activity against a negative control lacking a cloned origin (pCER19). RepDE or RepDN was diluted to the concentrations indicated and incubated with negatively supercoiled DNA as shown. Products were separated by electrophoresis in the presences of 1 µg/ml ethidium bromide. SC, negatively supercoiled substrate; OC, nicked open circular intermediate; RC, relaxed, covalently-close product. **b** RepDE and RepDN fusion proteins display magnesium-dependent cleavage and covalent linkage to single-stranded oligonucleotides derived from the replication origin. Rep proteins were incubated in the absence and presence of oligonucleotide and $MgCl_2$ as shown before separation by SDS-PAGE. Rep, position of free Rep monomer; Rep*, position of Rep-DNA covalent adduct. **c** RepDE and RepDN fusion proteins display specific binding to cloned origin sequences, distinct from non-specific binding to the cloning vector. Plasmids pCER*oriE*, pCER*oriN* and pCER19 were cut with PvuII to release target fragments containing cloned *ori* fragments of 353 bp (or an origin-free fragment of 322 bp from pCER19), plus a vector fragment of 2742 bp in all cases which serves as an intensity control. RepDE or RepDN was incubated with DNA from each of the three digests in the buffer lacking Mg^{2+} before separation by electrophoresis in the absence of ethidium bromide. Free, unbound target fragment of 322-353 bp; n/s, position of non-specifically bound fragment; Bound, position of specific Rep:*ori* complex; Control; residual vector DNA of 2742 bp.

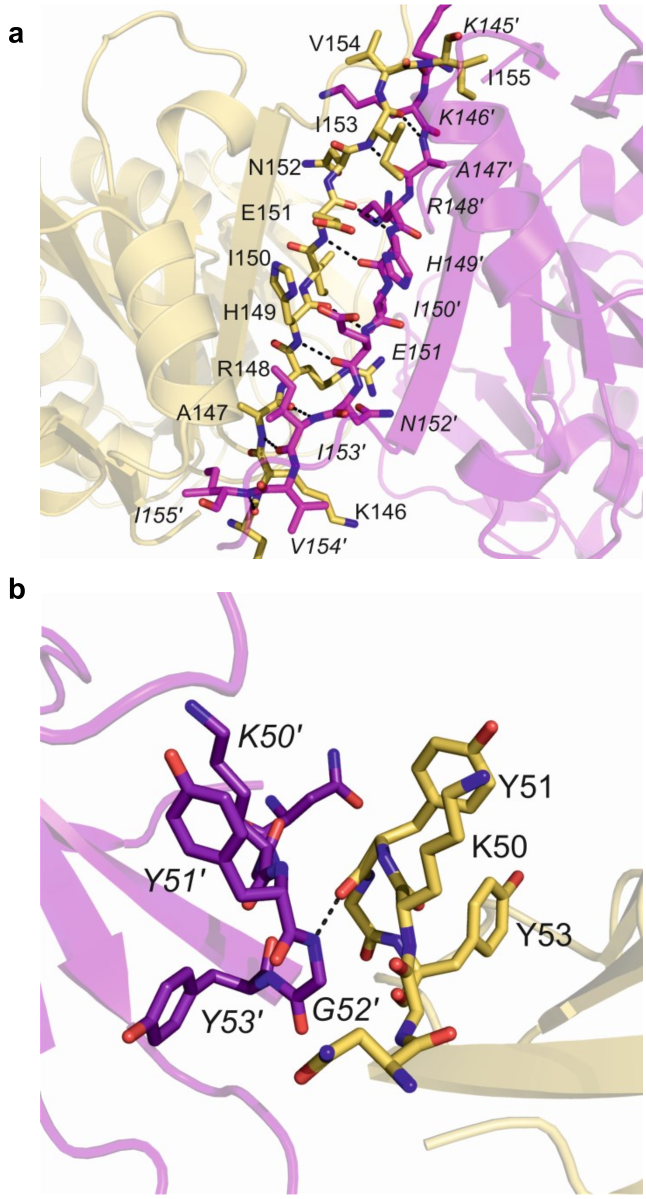

Figure S2 – Interfaces stabilising the ring conformation of RepSTK1

a The interface formed between strand β8 (residues 145-155) of each subunit is stabilised by multiple polar and hydrophibic interactions. **b** The second interface is formed by the tips of two loops and is stabilised by a single hydrogen bond.

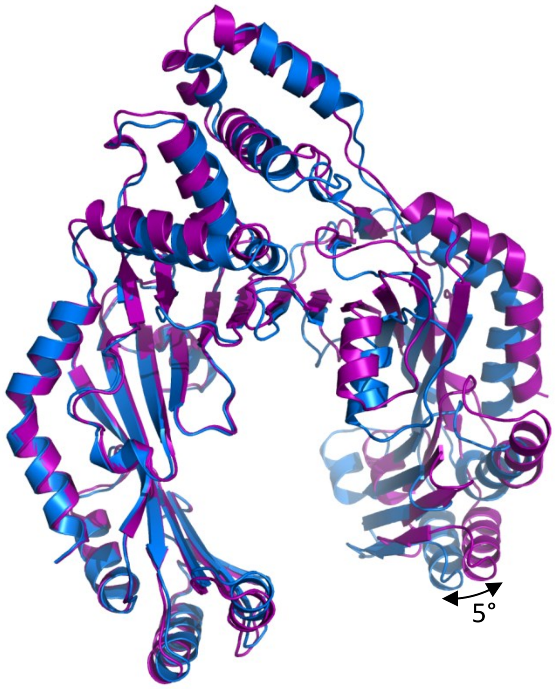

Figure S3 – Conformational flexibility in the catalytic domain of RepDE and RepDN

Superposition of the structures of RepDE (purple) and RepDN (blue) showing evidence of conformational flexibility within the catalytic domain of the *Staphylococcal* Rep proteins. The motion corresponds to a rigid body rotation of approximately 5°.

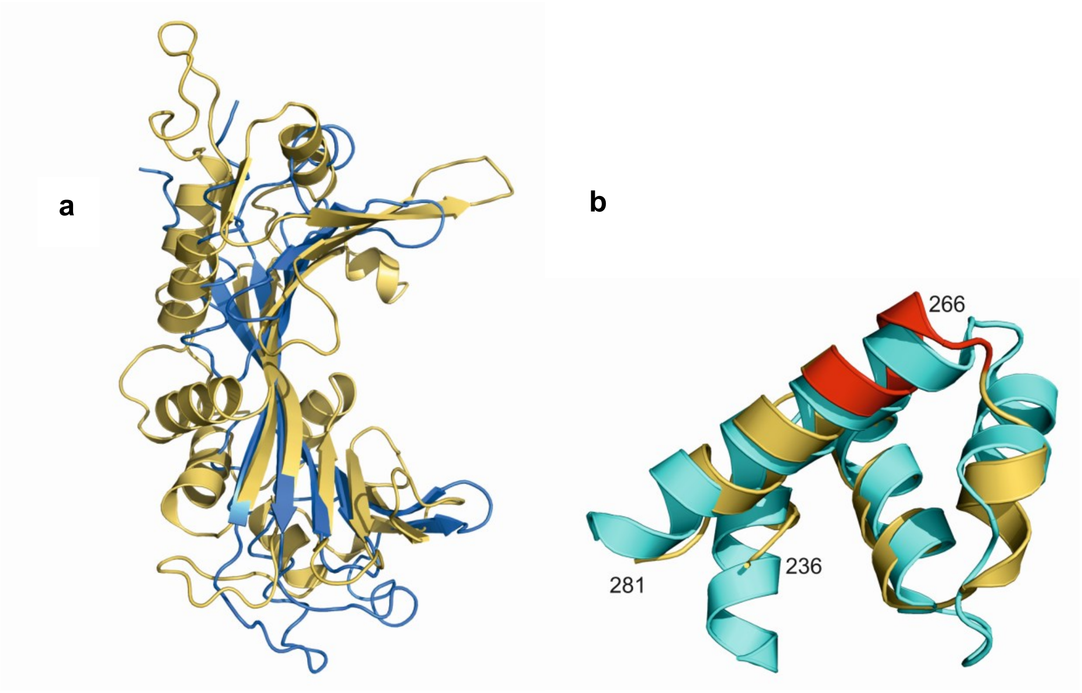

Supplementary figure S4 - Comparison of RepSTK1 and RepDE with known structures.

a Secondary structure based superposition of the RepSTK1 protein (gold) with TATA-binding protein (blue - pdb 1d3u). **b** Secondary structure based superposition of the DNA-binding domain of RepDE with an example RNA polymerase sigma factor (*S. aureus* SigA, pdb 4g6d). The DNA-binding domain is coloured gold with the DNA interaction site highlighted in red and the sigma factor coloured blue.

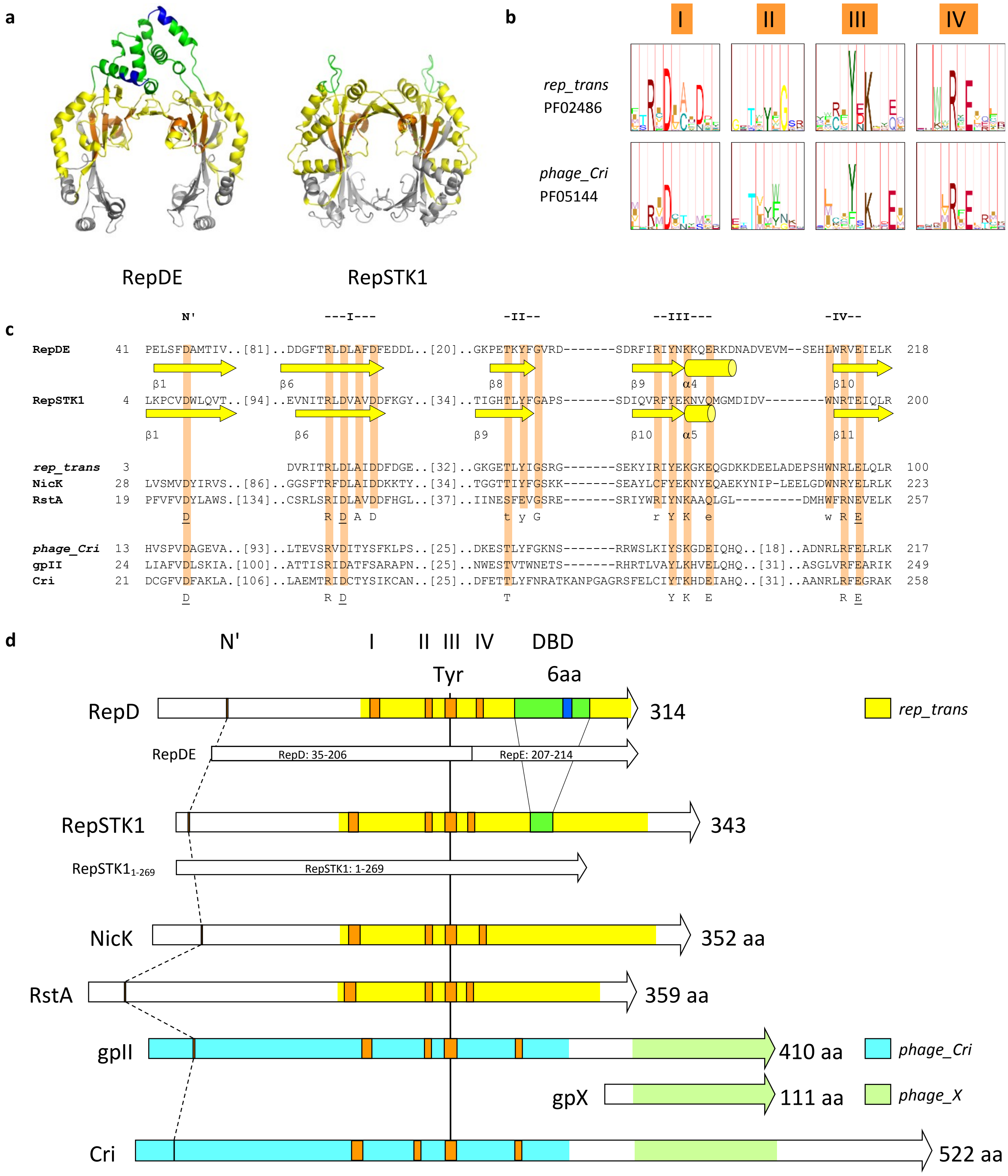

Supplementary figure S5 - Alignment of active site residues with rep_trans and phage_Cri motifs

a Yellow: *rep_trans* extent; orange: active site motifs; green: dimerisation/DNA-binding domain (DBD) or equivalent; blue: 6 amino acid specificity motif (6aa). **b** Signature motifs of *rep_trans* (PF02486) and *phage_Cri* (PF05144) families. HMM logos were adapted from the Pfam web site (http://pfam.xfam.org). Motifs are numbered **I**-**IV**. **c** Sequence alignment of RepDE and RepDTK1$_{1\text{-}269}$ with other members of *rep_trans* and *phage_Cri* families. Regions surrounding motifs **I**-**IV** are shown, as well as the conserved N-terminal aspartate (N') involved in metal ion binding. Secondary structure is shown for RepDE and RepSTK1. Representative sequences are also shown for relaxases NicK (ICE*Bs1*; UniProt P96635) and RstA (CTX phage; O34630) as members of the *rep_trans* family and gp**II** (phage M13, P69547) and Cri (pTLC, O85210) for *phage_Cri*. Consensus sequences for *rep_trans* and *phage_Cri* were taken from the corresponding entries in the Conserved Domain database (http://www.ncbi.nlm.nih.gov/cdd/). The N-terminal motif (N') is not part of any overlap between *rep_trans* and *phage_Cri*, and is suggested based on local sequence alignment and secondary structure prediction. Conserved amino acids within each motif are indicated; the acidic residues involved in metal ion coordination are underlined. **d** Location of motifs N', **I**-**IV** across representative reading frames. Examples are as given in (c); the extent of *rep_trans* and *phage_Cri* is indicated by shading. The extent of the fusion protein RepDE and 269 aa fragment RepSTK1$_{1\text{-}269}$ are indicated against their wild-type counterparts. DBD and 6aa regions of RepD and RepSTK1 are coloured green and blue as in (a). Also shown is the location and extent of M13 gpX, encoded in-frame with gp**II**, and the corresponding Pfam motif *phage_X* (PF05155) identified also within Cri.

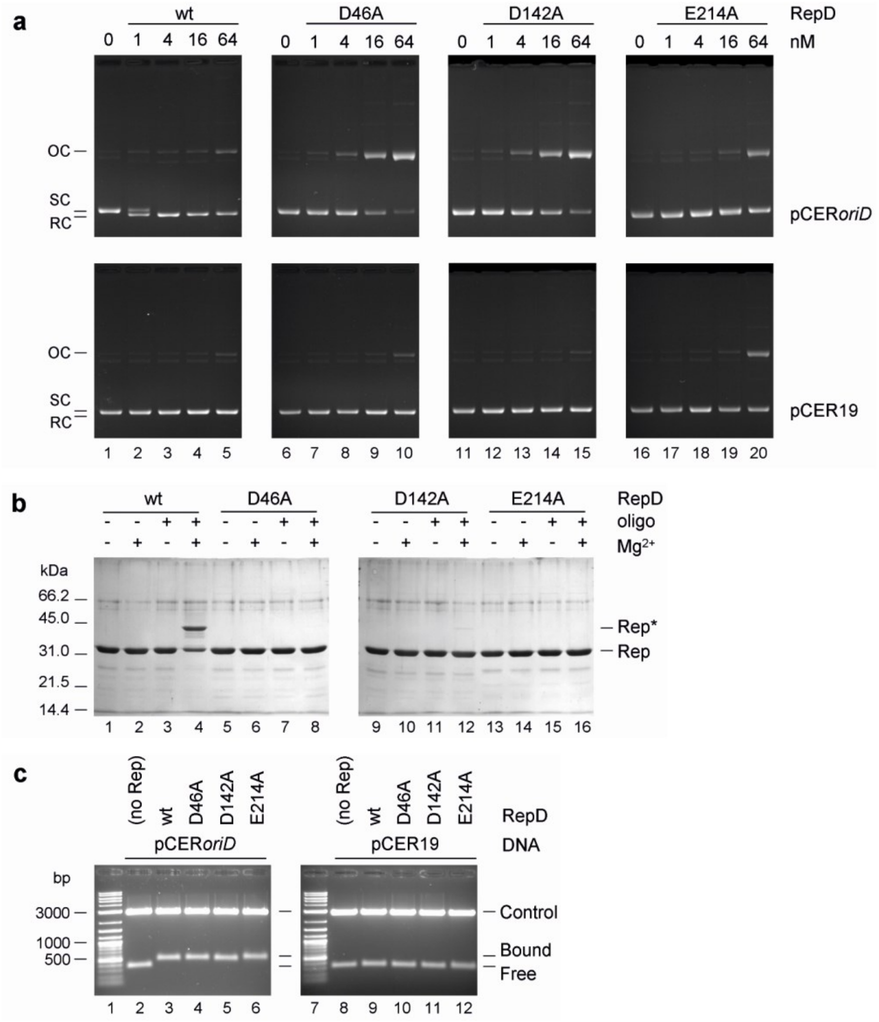

Figure S6 - Activity of RepD variants mutated at the metal binding site.

a Sequence-specific topoisomerase activity of RepD is lost in mutants D46A, D142A and E214A. RepD variants were diluted to the concentrations indicated and incubated with negatively supercoiled pCER*oriD* or pECR19 as shown. Products were separated by electrophoresis in the presences of 1 μg/ml ethidium bromide. SC, negatively supercoiled substrate; OC, nicked open circular intermediate; RC, relaxed, covalently-close product. None of the mutants were capable of forming an relaxed, covalently-closed product with pCER*oriD*. Some sequence-specific nicking remained at higher concentrations for D46A and D142A; residual nicking by E214A is non-specific, as a similar magnitude of nicking was observed against pCER19. **b** Mutations at the metal-binding site abolish oligonucleotide cleavage and adduct formation. RepD variants were incubated in the absence and presence of oligonucleotide and $MgCl_2$ as shown before separation by SDS-PAGE. Rep, position of free Rep monomer; Rep*, position of Rep-DNA covalent adduct. In comparison with the comparable amounts of free Rep protein and Rep* adduct obtained for the wild-type (lane 4), only a trace of adduct was evident for D142A (lane 12) and none at all for D46A and E214A (lanes 8, 16) despite heavy staining. **c** Mutations at the metal-binding site do not interfere with non-covalent binding at *oriD*. Plasmids pCER*oriD* and pCER19 were cut with PvuII to release target fragments containing cloned *oriD* (353 bp) or an origin-free vector fragment (322 bp), plus a vector fragment of 2742 bp in both cases which serves as an intensity control. Variants of RepD were incubated with DNA from these digests in a buffer lacking Mg^{2+} before separation by electrophoresis in the absence of ethidium bromide. Free, unbound target fragment of 322-353 bp; Bound, position of specific Rep:*ori* complex;

Control; residual vector DNA of 2742 bp. No significant difference was observed in the shift patterns of the variants. RepD does not form a non-specific complex with DNA from pCER19 under these conditions.

Experimental protocols detailed in extended materials and methods.

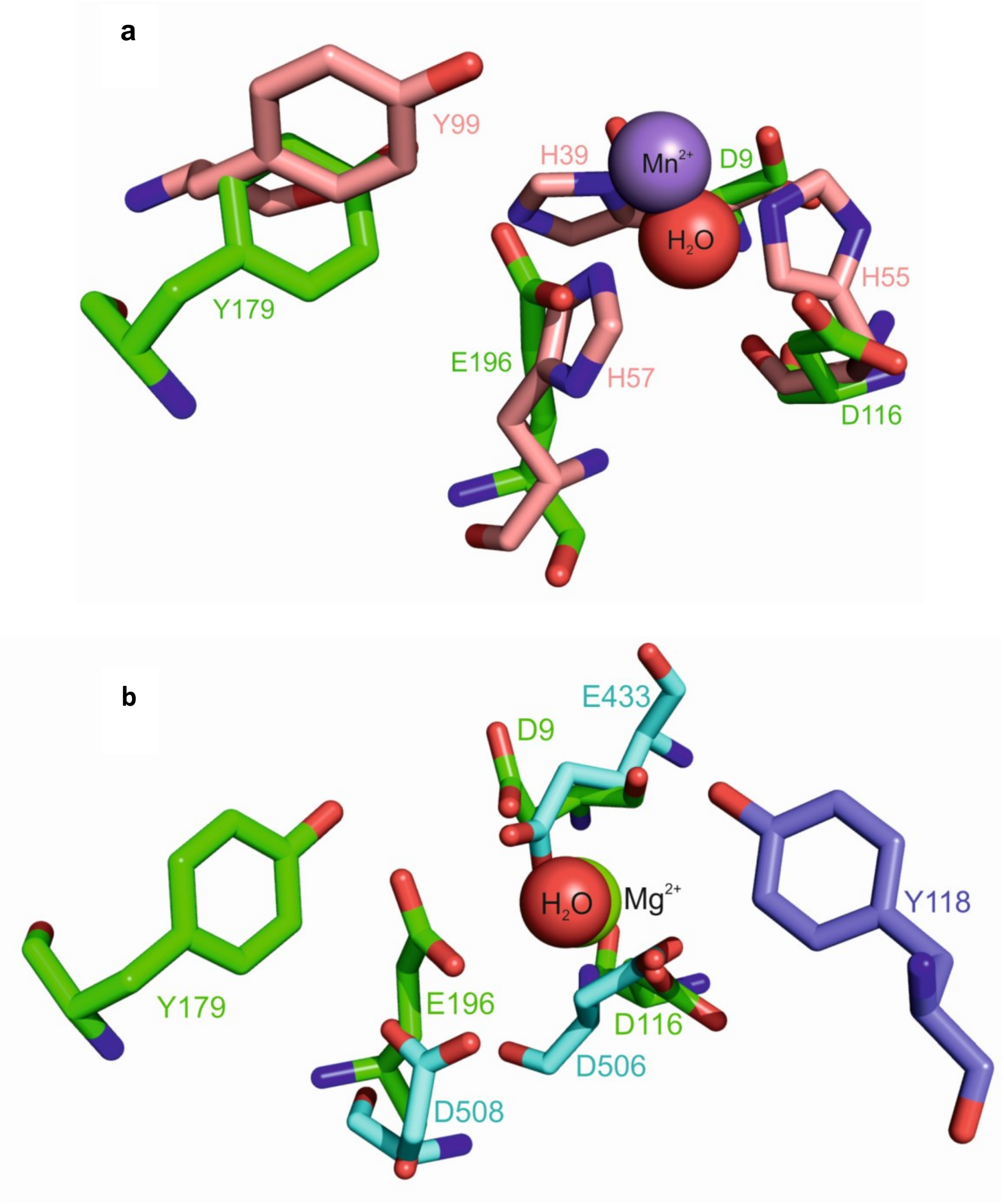

Supplementary Figure S7 - Comparison of the Active sites from Rep_trans with HuH relaxase and type II Topoisomerases.

a Superposition of the Cα positions of the metal coordinating amino acids allows a comparison of the active sites of RepSTK1 with RepB, a HUH family relaxase. Catalytic residues from RepSTK1 are coloured green and the water molecule in the metal binding site shown as a red sphere. Active site residues of RepB are coloured pink and the manganese ion coloured purple. **b** Comparison of the active sites of RepSTK1 with topoisomerase II based on alignment of the Cα atoms of the metal coordination residues. The active site residues from RepSTK1 are again coloured green. The metal binding amino acids from the TOPRIM domain of the Topoisomerase IIB subunit coloured cyan and the catalytic tyrosine from the Gyrase domain of the topoisomerase IIA subunit is coloured blue. The magnesium ion from the topoisomerase active site is coloured green.

Extended Materials and Methods

Construction of expression vectors for RepDE and RepDN:

Staphylococcal Rep proteins RepDE and RepDN are based on the 34 kDa variant of RepD (Thomas *et al*., 1995) with residues numbered according to the aligned sequences of members of the pT181 family (Projan & Novick, 1988).

The coding sequence for 34 kDa RepD (Met:F35-K314) was rebuilt in three sections following PCR amplification from plasmid pC221*cop903* (Projan *et al*., 1985) using primer pairs F35+/P-, P+/S- and S+/Ter- (Table S1). These fragments were combined via the silent PstI and SacI restriction sites (at L78-Q79 and E216-L217 respectively) while cloning into M13mp18 to yield vector M13mp18-235, in which the 34k reading frame is bounded by sites for AccI and EcoRI. The AccI-EcoRI fragment (equivalent to bases 1304-2137 from pC221, Genbank accession X02166) was subcloned into vector pHD (Thomas *et al*., 1990) via a compatible ClaI-EcoRI digest to form vector p235.

Derivatives of p235 were subsequently amplified using primers Nde+/Bam- prior to cloning in vector pET11m (a variant of pET11a lacking EcoRI, ClaI and HindIII sites) via NdeI and BamHI, creating the expression vector pET11m-235. The coding sequence for RepDE was created by amplification of the C-terminal region (L217-E314) of RepE from plasmid pS194 (Projan *et al*., 1988) using primers ES+/EE- (equivalent to bases 4021-4315 from pS194, Genbank accession X06627). This fragment was substituted into the vector pET11m-235 via SacI and EcoRI sites to yield the expression vector pET11m-RepDE.

The coding sequence of RepDN was created by amplification of the C-terminal region (L217-K314) of RepN from plasmid pCW7 (Balson and Shaw, 1990) using primers S+/Ter- (equivalent to bases 850-1143 from pCW7 fragment, Genbank accession J03323), before substituting this fragment via SacI and EcoRI sites within M13mp18-235 to yield vector M13mp18-237. The complete reading frame was re-amplified using primers Nde+/RPDNREV before cloning into pET11a (Novagen) via Nde and BamHI sites to yield expression vector pET11a-RepDN.

Construction and purification of substrate plasmids for activity assays

Plasmids pCERoriE and pCERoriN were constructed as substrates for RepDE and RepDN respectively as described previously for pCERoriD (Machon *et al.* 2010). Oligonuceotides oriE+/- and oriN+/- (Table S1) corresponding to the origin of replication recognised by RepDE and RepDN were mixed in an equimolar ratio and annealed by heated to 95°C for 5 minutes followed by slow cooling to 4°C. The duplex DNA product was cloned into pCER19 via HindIII/XmaI restriction sites and the resulting plasmid was used to transform *E. coli* strain *DH5α.* Positive transformants were used to inoculate 1L volumes of LB broth (containing 100 µg/ml Ampicillin) and cells were grown to stationary phase. Negatively supercoiled DNA was purified by alkaline lysis followed by 2 rounds of CsCl/ethidium bromide density gradient centrifugation.

Site directed mutagenesis

Mutagenesis of the putative metal binding residues was performed using the Quik-change protocol (Stratagene, La Jolla, Ca), with primer pairs D46A+/-, D142A+/- and E214A+/- (Table S1) to alter the 34 kDa variant of RepD cloned in pET11.

Topoisomerase assays

Rep protein was diluted to 64, 16, 4, 1, 0.25 nM in 30/28x K200 buffer (53.6 mM Tris.HCl pH7.5, 214.3 mM KCl, 1.07 mM EDTA, 10.7 mM $MgCl_2$, 10.7 % v/v ethanediol) and 28 µl aliquots mixed with 2 µl of supercoiled (SC) plasmid DNA at 0.25 mg/ml in TE buffer to give a final buffer concentration of 50 mM Tris.HCl pH7.5, 200 mM KCl, 1mM EDTA, 10 mM $MgCl_2$, 10 % v/v ethanediol. Samples were

incubated for 60 minutes at 30°C when the reaction was quenched by the addition of 4 µl stop solution (0.25 M EDTA, 50 % v/v glycerol, 0.05 % w/v bromophenol blue) before loading on to a 1 % w/v agarose gel cast in TBE buffer containing 1 µg/ml ethidium bromide. Electrophoresis was performed at 5 V/cm, 450 Vh. All protein dilutions were calculated per dimer of RepD.

Oligonucleotide cleavage assays

Rep protein was diluted to 0.4 mg/ml in K400 buffer (50 mM Tris.HCl, pH 7.5, 400 mM KCl, 1 mM EDTA, 10% v/v ethanediol). Aliquots of 10 µl Rep (4 µg) were combined with 5 µl of 40 mM $MgCl_2$ or water, to which 5 µl of oligonucleotide CT23 (118 µM) dissolved in TE buffer was added in order to start the reaction. Samples were incubated at 30°C for 60 minutes after which the reaction was stopped by the addition of an equal volume of 2x SDS loading buffer. All samples were heated to 95°C for 5 minutes before separation by SDS-PAGE (12 % polyacrylamide). Bands were visualised by staining with Coomassie Blue G250.

DNA binding (mobility shift) assays

Plasmid pCERoriE or pCERoriN was digested to completion with PvuII. Protein-DNA complexes were formed by mixing 1 µg DNA with 100 nM RepDE or RepDN dimer respectively, in 50 mM Tris.HCl, pH 7.5, 200 mM KCl, 1 mM EDTA, 10 % v/v ethanediol. The final reaction volume was 30 µl. Samples were incubated for 60 minutes at 30°C after which 4 µl stop solution (0.25 M EDTA, 50 % v/v glycerol, 0.05 % w/v bromophenol blue) was added. Samples were analysed by electrophoresis in 1.2 % w/v TAE agarose at 5 V/cm for 2 hours. Bands were visualised by staining with 1 µg/ml ethidium bromide.

Supplementary Table S1 - Oligonucleotide sequences

Name	Sequence (5'→3')
F35+	GGGAA GCTTG TCGAC T**ATG**T TTTTT ACCAC CCCTC
P-	GGGCT GCAGT GCTTT AGCTT TAAAC
P+	GGGCT GCAGG AAAAG GTTTA TATCG
S-	CCCGA GCTCA ATTTC TACAC GCC
S+	CCCGA GCTCA AAAGA GATAT GGTTG
Ter-	GGGGA ATT**CT A**CTTC CAAAA TCTAA ATTC
ES+	GGGAT TGAGC TCAAA AGAGA TATGG TTGAT TATTG G
EE-	CCGGA ATTCT ACTCC CAAAA TCTAA ATTCA CG
Nde+	GGAAT TCCAT ATGTT TTTTA CCACC CC
Bam-	CGGGA TCCTT GCGGC TTTGG C
RPDNREV	CGGGA TCCGA ATTCT ACTTC CAAAA TC
CT23	CTAAT AGCCG GTTAA GTGGT AATTT
oriE+	AGCTT ACTCA AATTT TTCTA AAACC GGATA CTCTA ATAGC CGGTT AAACC GACAT ATTAT GTACA C
oriE-	CCGGG TGTAC ATAAT ATGTC GGTTT AACCG GCTAT TAGAG TATCC GGTTT TAGAA AAATT TGAGT A
oriN+	AGCCT TAGTC AATTT TTCTA AAACC GGCTA CTCTA ATAGC CGGTT AAACC GACAT ACTATGTACA C
oriN-	CCGGG TGTAC ATAGT ATGTC GGTTT AACCG GCTAT TAGAG TAGCC GGTTT TAGAA AAATT GACTA A
D46A+	CAACC AGAAT TAAGT TTTGC GGCTA TGACA ATCGT TG
D46A-	CAACG ATTGT CATAG CCGCA AAACT TAATT CTGGT TG
D142A+	GGTTT TACGA GGTTA GCGTT AGCTT TTGAT TTTGA AG
D142A-	CTTCA AAATC AAAAG CTAAC GCTAA CCTCG TAAAA CC
E214A+	CATTT ATGGC GTGTA GCGAT TGAGC TCAAA AGAGA TATG
E214A-	CATAT CTCTT TTGAG CTCAA TCGCT ACACG CCATA AATG

All oligos were obtained from Eurofins Genomics, Ebersberg, Germany.

Supplementary References

Balson, D.F. and Shaw, W.V. (1990) Nucleotide sequence of the *rep* gene of staphylococcal plasmid pCW7. Plasmid 24:74-80.

Machon, C., Lynch, G.P., Thomson, N.H., Scott, D.J., Thomas, C.D. and Soultanas, P. (2010) RepD-mediated recruitment of PcrA helicase at the Staphylococcus aureus pC221 plasmid replication origin, oriD. *Nucleic Acids Res.* **38**:1874-1888.

Projan, S.J., Kornblum, J., Moghazeh, S.L., Edelman, I., Gennaro, M.L. and Novick, R.P. (1985) Comparative sequence and functional analysis of pT181 and pC221, cognate plasmid replicons from *Staphylococcus aureus*. Mol. Gen. Genet. 199:452-464.

Projan, S.J. and Novick, R. (1988) Comparative analysis of five related Staphylococcal plasmids. Plasmid 19:203-221.

Projan, S.J., Moghazeh, S. and Novick, R.P. (1988) Nucleotide sequence of pS194, a streptomycin-resistance plasmid from *Staphylococcus aureus*. Nucleic Acids Res. 16:2179-2187.

Thomas, C.D., Balson, D.F. and Shaw, W.V. (1990) *In vitro* studies of the initiation of staphylococcal plasmid replication. Specificity of RepD for its origin (*oriD*) and characterization of the Rep-*ori* tyrosyl ester intermediate. J. Biol. Chem. 265:5519-5530.

Thomas, C.D., Nikiforov, T.T., Connolly, B.A. and Shaw, W.V. (1995) Determination of sequence specificity between a plasmid replication initiator protein and the origin of replication. J. Mol. Biol. 254:381-391.

Schuster-Böckler, B., Schultz, J. and Rahmann, S. (2004) HMM Logos for visualization of protein families. BMC Bioinformatics **5**:7.
